# Dynamics of the Oxygen Atom Transfer Reaction between Carbon Dioxide and the Tantalum Cation

**DOI:** 10.1021/acs.jpclett.3c01078

**Published:** 2023-06-08

**Authors:** Marcel Meta, Maximilian E. Huber, Tim Michaelsen, Atilay Ayasli, Milan Ončák, Roland Wester, Jennifer Meyer

**Affiliations:** † Fachbereich Chemie und Forschungszentrum OPTIMAS, RPTU Kaiserslautern-Landau, Erwin-Schrödinger Straße 52, 67663 Kaiserslautern, Germany; ‡ Institut für Ionenphysik und Angewandte Physik, Universität Innsbruck, Technikerstraße 25, 6020 Innsbruck, Austria

## Abstract

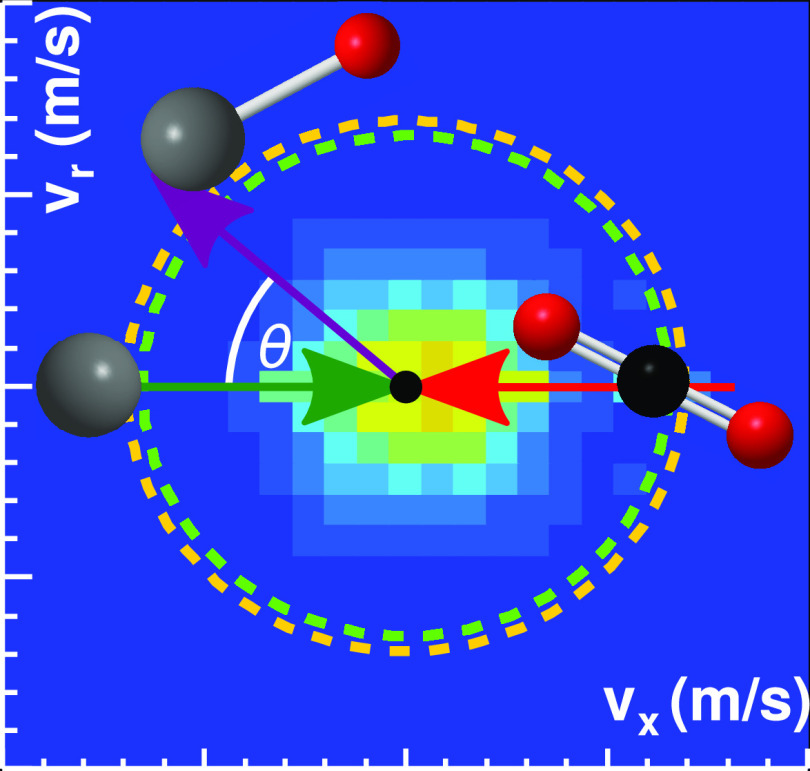

The understanding of fundamental atomic-level processes often requires well-defined model systems. The oxygen atom transfer from CO_2_ to a transition metal cation in the gas phase presents such a model system. We investigate the reaction of Ta^+^ + CO_2_ for which the formation of TaO^+^ is highly efficient and attributed to multistate reactivity. Here, we study the atomistic dynamics of the oxygen atom transfer reaction by recording experimental energy and angle differential cross sections by crossed beam velocity map imaging supported by *ab initio* quantum chemical calculations. Product ion velocity distributions are dominated by signatures for indirect dynamics, despite the reaction being highly exothermic. Product kinetic energy distributions show little dependence on additional collision energy even with only four atoms involved, which hints at dynamical trapping behind a submerged barrier.

Designing and controlling chemical reactions at the molecular level is an aim chemists strive to achieve. Complex reactions are broken down to model systems and elementary reaction steps studied to determine intrinsic properties of a reaction mechanism or reactive center.^1−3^ One approach is to transfer the reaction into the gas phase, which presents an environment void of any unwanted interactions. These systems are not directly comparable to, for example, condensed phase catalytic systems, but can help to determine structure reactivity relationships and serve as benchmarking systems for theory.^4^ The reactions of transition metals in the gas phase are a classic example of such experiments. Over the last decades, a wide range of reactants like ions, clusters, and nano particles up to metal organic complexes have been under investigation by a similar wide range of methods, ranging from kinetics under single and multiple collision conditions to spectroscopy over a wide spectral range and time scales.^3,5−13^ However, at the heart of chemical reactions are reactive collisions, and therefore, dynamical aspects have to be included to obtain the full picture. Relying only on arguments based on stationary properties can be misleading.^14^ Experimental energy and angle differential cross sections offer a view into the atomistic dynamics of a reactive collision, that is how atoms rearrange during the reactive collision itself.^15−20^ By interpreting the velocity distributions and identifying dynamic fingerprints, we can derive information about the atomic level rearrangement during the reaction and energy partitioning between translation and internal degrees of freedom. Weisshaar and co-workers investigated the reaction dynamics of transition metal ions with hydrocarbon molecules in the early 2000s. However, the experiment focused on cobalt and nickel cations in reactions with C_3_–C_4_-hydrocarbons.^21,22^ Today, studies on the dynamics of ion molecule reactions are limited to a small number of experiments.^23−27^ However, none of these focus on transition metal chemistry. Here, we present results from our new experimental crossed beam imaging setup. We studied the reaction of the tantalum cation with carbon dioxide (reaction 1) and present experimental product ion velocity distributions from which we extract information about the atomistic dynamics and thereby complement the literature on this reaction.^28−32^
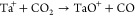
1The controlled reduction of carbon dioxide by oxygen atom transfer to another reactant moved into focus due to carbon dioxide being abundant and one of the primary green house gases. However, the strong C=O bond presents a significant barrier to any activation reaction. Over the last few decades, transition metal ions and clusters have been studied with respect to their CO_2_ activation using kinetic and spectroscopy methods. If one reduces the model system down to a single atom/ion, the number of transition metal cations showing an exothermic oxygen atom transfer is limited.^5,29^ The most reactive elements are located in the 5d-series. Tantalum is one of these elements. Considerably cheaper and more abundant than platinum or iridium, it is an alternative to the established elements.^10,30,33,34^ The reaction was found to efficiently happen at room temperature even though the reaction on the ground state surface of the Ta^+^ is associated with a barrier of about 0.5 eV^28−30,33^ (Figure 1).

**Figure 1:**
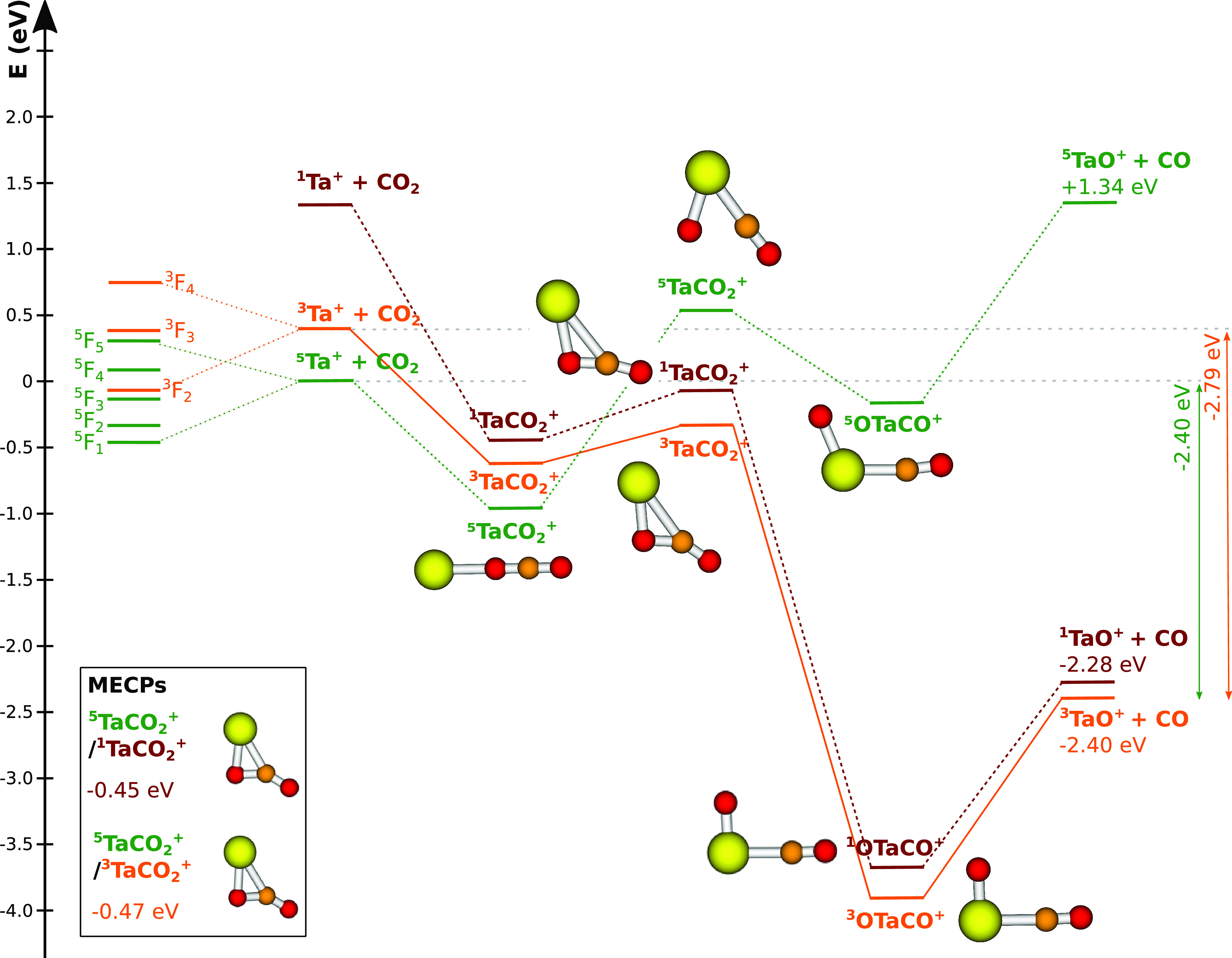
Reaction coordinate for Ta^**+**^ + CO_2_ →TaO^+^ + CO. Structures of the stationary points along the reaction coordinate, i.e., for the pre-reaction and post-reaction complexes as well the transition states, for the quintet surface (green), triplet (orange), and singlet (red) are given (Ta = yellow, carbon = orange, oxygen = red; structural data can be found in the ESI). The given reaction energies of *E*(^5^Ta^+^) = −2. 40 eV and *E*(^3^Ta^+^) = −2. 79 eV are used for calculating kinematic cut-offs. Blue arrows indicate the investigated relative collision energies. Calculated at the CCSD(T) level with optimization at B3LYP and CCSD levels (see the Methods for details). The minimum energy crossing points (MECPs) were optimized at the CCSD level.

The reaction between Ta^+^ and CO_2_ is highly exothermic if one assumes a crossing of spin surfaces during the reaction, i.e., a reaction from the quintet ground state of the Ta^+^ to the triplet ground state of TaO^+^ or to an excited singlet state of the TaO^+^ during which the reaction coordinate has to switch spin surfaces. This crossing of the reaction coordinate to a different spin state, which is referred to as multistate reactivity, is established for the present system.^29,31,35^ Considering the experimental rate constants, which are on the order of the Langevin rate,^28−30^ the passing through one of the crossing points has to be highly efficient. The first electronically excited state of the Ta^+^ is a triplet state about 0.4 eV above the quintet ground state.^36^ Due to the strong spin–orbit coupling, the total spin *S* is no longer a good quantum, and we should consider the total angular momentum *J*. The quintet manifold is shown in Figure 1 and spans about 0.8 eV crossing with the lowest lying state of the triplet manifold.

The spin–orbit coupling which mediates the spin-crossing, also leads to coupling between the quintet and triplet atomic states.^37^ Stachowka et al. found that the ground state has only about 5–10% triplet mixed character (*J* = 1–4 about 5% and 10% for *J* = 5). Unfortunately, a full *ab initio* treatment for the whole potential energy landscape, including all relevant electronic states, is beyond the scope of the present study.

Starting from the triplet state ^3^Ta^+^, the reaction shows all of the features common to an exothermic gas phase ion molecule reaction. A pre-reaction complex is formed which passes over a submerged transition state into a post-reaction well from which the free products are formed (Figure 1). The crossing point is located between the pre-reaction minimum and the transition state. In previous calculations, its energy relative to the entrance channel shows a large spread.^31,38^ We find both possible crossing points at approximately −0.4 to −0.5 eV with respect to the ground state reactants and thus to be submerged (Figure 1). This is in line with the experimental reaction rates at room temperature. The reaction moves from a colinear pre-reaction complex, where the tantalum coordinates to one of the oxygen atoms, over to a bent structure. During the bending of the carbon oxygen bond, one of the crossing points can be passed through. Comparing structures it becomes obvious that the structures at the crossing points closely resemble that of the triplet transition state including energetics. Once the carbon oxygen bond is broken, CO is coordinated to the TaO^+^ and subsequently leaves.

The experimental energy and angle differential cross sections for the title reaction for three relative collision energies are presented in Figure 2. A beam of Ta^+^ ions has been crossed with a molecular beam of carbon dioxide under single collision conditions. Details about the experimental setup are given in the methods sections. The first row of Figure 2a–c shows the normalized product ion velocity distributions. The TaO^+^ ions are predominantly isotropically scattered around the center-of-mass. Superimposed onto the distributions are rings which represent the kinematic cut-offs for reactions of ^5^Ta^+^ (green) and ^3^Ta^+^ (orange) ions. The kinematic cut-off corresponds to the maximum velocity of the TaO^+^ ions reach under energy and momentum conservation. It is defined by the energetics of both reactant beams and the reaction exothermicity. We observe a small asymmetry toward the backward hemisphere at the lowest collision energy. Upon increasing the collision energy from 1 to 2 eV, the distribution shifts toward scattering into the forward hemisphere. A more quantitative depiction of the shift toward forward scattering can be seen in the integrated angular distributions (Figure 2a–c). The kinematic cut-off for the singlet state as well as the formation for ^5^TaO^+^ which is energetically feasible at the two higher collision energies (see Figure S2).

**Figure 2:**
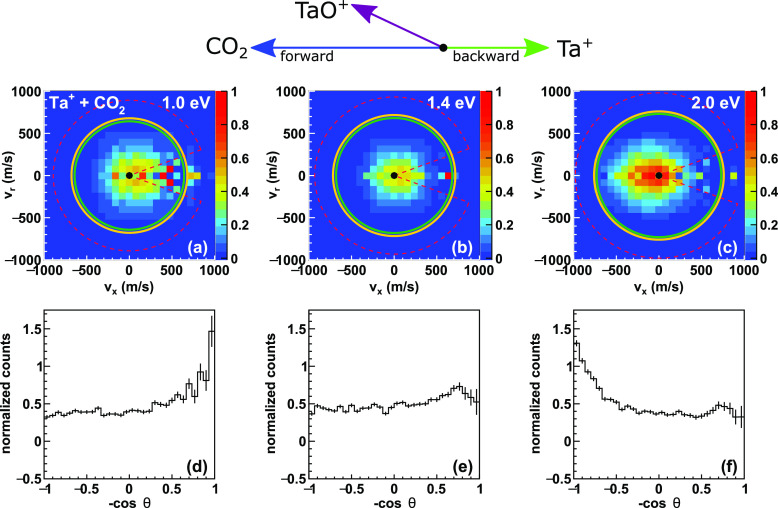
Experimental differential cross sections. (a–c) Velocity distributions of the TaO^+^ product ion at three collision energies in the range of 1–2 eV. The superimposed circles represent the kinematic cut-off for reaction of Ta^+^ in its electronic ground state (^5^Ta^+^, green) and the first electronically excited state (^3^Ta^+^, orange). The singlet state has been omitted for clarity. A simplified Newton diagram is given at the top. At a scattering angle at or close to θ = 180°, residual hits of the incident ion beam are visible (for details, see the Methods section). The angular integrated distributions are shown in the second row (d–f). They confirm the slight asymmetry already visible in the velocity distributions. The significant backward scattered events for the two lower collision energies stem from residual contributions from the reactant ion beam. The red lines indicate a cut in velocity space for the evaluation of the respective energy distributions.

Here, we stress that we cannot directly determine the electronic state distributions within the Ta^+^ beam formed in the laser vaporization source. Our ions undergo on average about 10^3^ collisions with helium before exiting the immediate source region which is insufficient for significant quenching exited states.^39−41^ Taking the available literature on different laser vaporization sources,^42−45^ we assume approximately 10–20% electronically excited state (^3^F and ^3^P) in the ion beam which corresponds to a thermal plasma of about 3000 K. Boltzmann statistics of the electronic state distributions for different plasma temperatures are given in the SI (see Table S2). Our experimental resolution in combination with the highly indirect nature of the reaction does not allow us to draw any conclusion from the experimental directly as all events are scattered well within the kinematic cut-off associated with the ground state (^5^F_1_).

A direct comparison of the internal energy and product kinetic energy as a function of the collision energy is given in Figure 3. The internal energy distributions shift toward higher energies, whereas the kinetic energy distributions are near constant (Figure 3d). At all collision energies, a large fraction of the available energy is partitioned into internal excitation and additional collision energy is also almost completely partitioned into internal energy. The fraction of available energy partitioned into internal excitation is about 60% at all collision energies (Table 1).

**Figure 3:**
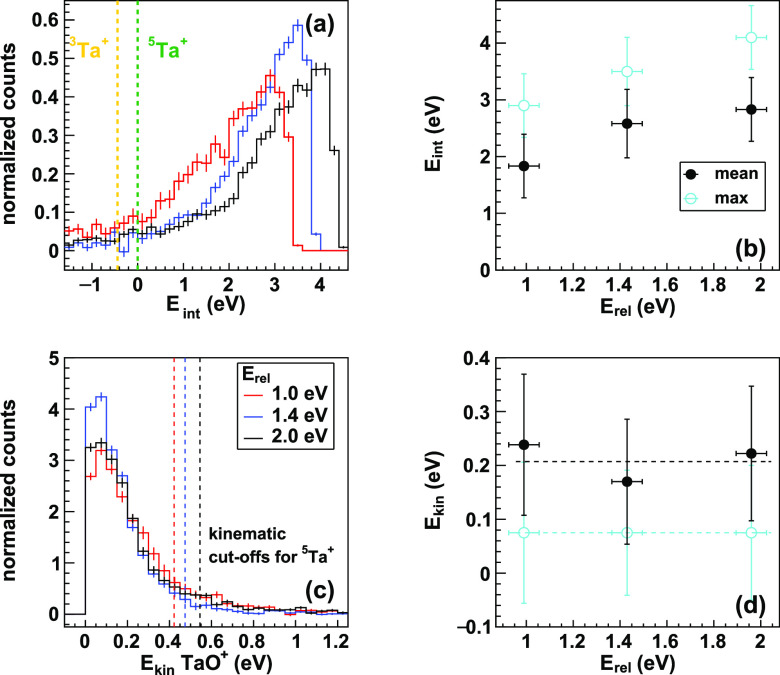
Internal and product kinetic energy distributions. The first column gives a comparison of the energy partitioned into internal excitation (a) and (c) and kinetic energy into the motion of the TaO^+^. Panels (b) and (d) give the mean energies of the respective distributions (black, closed circle) and the bin with the maximum intensity (cyan, open circle). Additional collision energy is almost completely channeled into internal excitation. The kinetic energy distributions are almost the same at all three collision energies. The dashed lines in (b) and (d) are fits to data. The error bars illustrate 1σ-errors of the respective energy distributions as by Gaussian error propagation from the input beams, in which the energy spread is given as well as the 1σ-error.^47^ All distributions are calculated from the hits within a pie cut, as indicated by the pink dashed lines in Figure 2, which lack contribution from the residual ion beam. In Figure S1, comparisons to the full distributions are given.

**Table 1:** Fraction of Available Energy *f*_int_ Partitioned into Internal Excitation^a^

*E*_rel_ (eV)	mean *E*_int_ (eV)	*f*_int_
1.0	1.83	0.53
1.4	2.58	0.67
2.0	2.83	0.65

a Total available energy given by *E*_rel_ plus reaction from ^5^Ta^+^ + CO_2_ →^3^TaO^+^ + CO.

Isotropic scattering around the center-of-mass is a signature of indirect atomistic dynamics. The two reactants form a long-lived complex whose lifetime exceeds the rotational period of the complex. The complex subsequently decays statistically in all directions which in turn leads to isotropic scattering.^18,23^ During the lifetime of the complex, the kinetic energy of the collision is partitioned into rotational and vibrational degrees of freedom of the molecular products. With increasing collision energy the reaction becomes slightly more direct as TaO^+^ ions are scattered into the forward hemisphere into small scattering angles. This is surprising, as the heavy tantalum ion (*m* = 181 amu) needs to turn around for TaO^+^ to be scattered into the forward hemisphere. This requires a large momentum transfer akin to a direct rebound mechanism. The smaller angular range also indicates that the reactions require a colinear orientation because otherwise more high angle scattering should be observable. We, therefore, assume a colinear approach geometry which is in-line with the structures of the pre-reaction complexes found as stationary points. For all three theoretically investigated spin states, one of the oxygen atoms points toward the tantalum ion. The found scattering signatures for the indirect as well as the direct mechanism, are associated with small impact parameter collisions.^18,46^ For the present case, this finding is somewhat unexpected because for such small systems with thermal rates close to collision rate, chemical intuition suggests the reaction to be rather dominated by large impact parameter collisions.

In the present case, it is striking that additional collision energy is almost exclusively partitioned into internal excitation considering it is a four-atom reaction in which two diatomic molecules are formed. Hence, for each molecular product, only three degrees of freedom are available as heat bath: the stretching vibration and the two rotational modes. However, the high fraction of internal excitation and near constant product ion kinetic energy hint at a dynamic bottleneck along the reaction coordinate behind which the reaction is trapped and which in turn provides enough time for efficient energy transfer from translation to internal degrees of freedom. Dynamical trapping is known to occur for gas phase reactions of a few atoms even if barriers are submerged.^21,48−51^ Once the reaction passes through the bottleneck, a large fraction of the released energy is channeled into product translation. This agrees with the common assumption that a large amount of energy available in the exit channel leads to a rapid separation of the products.^52^ Here, we have to consider the total energy partitioned into product translation, denoted as *E*_rel_^′^, which is the kinetic energy of TaO^+^ and CO (Figure S2). On average, 1.4 ± 0.3 eV is channeled into product translation. Referring to the reaction coordinate, we have two possibilities for the potential bottleneck if we assume the reaction to swiftly transition through the post-reaction minimum or bypassing it altogether. The first option are the crossing points from the quintet to triplet or singlet surface, and the second option are the respective transition states. All are close in structure and energy and in all cases the reaction would be trapped in a pre-reaction well. If in a first approximation, we assume the reaction leads to TaO^+^ in its triplet ground state and simply add the average product kinetic energy of 1.4 ± 0.3 eV to the 2.4 eV energy release for ^3^TaO^+^ + CO formation. We end close to the pre-reaction minima of the quintet and triplet surface. This further supports our interpretation of the reaction being trapped on the reactant side of the barrier.

We presented experimental energy and angle differential cross sections for the oxygen atom transfer reaction between the tantalum cation and carbon dioxide. The velocity distributions of the TaO^+^ ion are mostly isotropic, revealing indirect dynamics to be dominant even at a collision energy of 2 eV and a reaction exothermicity of approximately 2.5 eV. The kinetic energy distributions of the products do not shift with increasing collision energy, meaning that additional energy is almost exclusively partitioned into internal excitation of the products. This is unexpected because only four atoms are involved and both molecular products are diatomic. Our experiments suggest a dynamic bottleneck along the reaction coordinate behind which the reactants are trapped. Once past or over the bottleneck energy is then partitioned into product translation. Comparison to the stationary points along the reaction coordinate suggests the reaction to be trapped in the pre-reaction well prior to crossing over to the triplet or singlet state with the quintet pre-reaction well being the most likely candidate. The colinear structure of the pre-reaction complex is supported by the appearance of scattering events in the forward hemisphere at low scattering angles at the highest investigated collision energy. These events can be attributed to a direct rebound mechanism due head-on collisions. To confirm our speculations, trajectory simulations for TaO^+^ + CO_2_ are needed, especially in light of the possible dynamic trapping and high fraction of internal excitation. Currently, such calculations are, however, highly challenging due to the high number of low-lying electronic states in the tantalum complexes. Experimentally, we will further investigate the oxygen transfer reaction by studying the reaction with niobium, for which similar electronic arguments have been used and which is the lighter homologue to tantalum. This will allow us to gain insight into the observed momentum transfer and thus the atomistic dynamics. The present study is the first from our new experimental setup which opens another window into the reactions of transition metal (cluster) ions and adds experimental atomistic reaction dynamics as a new tool to study these fascinating reactants.

## Methods

### Experiment

We combine crossed beams with 3D velocity map imaging to record energy and angle differential cross sections.^27,47,53^ We prepare the ion beam using a home-built laser vaporization source.^54,55^ We focus the second harmonic of a Nd:YAG laser (532 nm, 20 Hz, ≈ 4 mJ/pulse (5 × 10^7^ W/cm^2^)) onto a rotating tantalum foil and form a plasma. A synchronized helium pulse (8 bar of He, 40 μs) extracts the plasma perpendicular to the laser beam toward the interaction region. The ion beam is confined to an expansion channel of 61 mm length and a diameter of 2 mm. The ions undergo on the order of 10^4^ collisions with helium before entering the high vacuum region of the source chamber (≈ 5 × 10^6^ – 1 × 10^5^ mbar during operation). The number of collisions was estimated using the relative pressure increase of a single gas pulse, the Langevin collison rate of Ta^+^ + He and the maximum possible velocity of a supersonically expanding helium gas pulse. We have no direct control over the electronic state distribution within the ion beam but assume only significant contributions from the quintet ground state and electronically excited triplet states to be present because the first electronically excited singlet state is too far up in energy and statistically unfavorable^36^ (Table S2). The source is operated such that only Ta^+^ cations make up the ion beam, and cluster formation is suppressed. The ion source is oriented in line with the velocity map imaging spectrometer.^47,56^ The Ta^+^ beam is transferred into the interaction region of the velocity map imaging stack by using a set of deflectors and Einzel-lenses. Subsequently, it is characterized by velocity map imaging to adjust energy and angle. A potential bias to the source region relative to the bias of the velocity map imaging stack can be used to adjust the collision energy. The CO_2_ molecular beam is prepared using a home-built piezo valve and expanding pure carbon dioxide. Similar to the ion beam, the molecular beam is characterized by velocity map imaging after electron impact ionization. The 1σ-error of the collision energies due to velocity and angular spread of both input beams is between 65 to 140 meV for the presented experiments. The two beams are crossed at a relative angle of 150° and the products perpendicularly extracted toward a position and time sensitive detector. Product ions hit a multichannel plate phosphor screen combination, and a ccd-camera records the position of impact. A photo multiplier tube records the arrival time. Combing both data sets, we can extract all three velocity components and thereby recover the whole Newton sphere due to the cylindrical symmetry in the center-of-mass frame allowing us to recover thevelocity along *z*-direction perpendicular from the scattering plane from the time-of-flight using *v*_*y*_.^47^ Our data analysis follows the method established by Wester and co-workers.^27,47,56^ To plot our data and make it comparable to sliced images, we mapped the 3D Newton Sphere again onto a 2D representation plotting the product ion velocity distributions as 2D histograms using the center-of-mass velocity denoted *v*_*x*_ and the radial velocity *v*_*r*_ in the center-of-mass, which is recovered from *v*_*y*_ and *v*_*z*_ velocities. To account for the different solid angles the data shown in the 2D histogram is weighted by the radial velocity. Integrating the original distributions over energy or scattering angle yields the 1D histograms displayed in Figure 2.

### Theory

Transition metal complexes are difficult to model due to possible involvement of multireference effects and the multitude of low-lying electronic states including states of different spin multiplicity. Here, we used the single-reference coupled cluster singles and doubles (CCSD) method to optimize the clusters, with subsequent energy recalculation at the CCSD level with iteratively included triples (CCSD(T)) level. Transition states were optimized at the B3LYP level, and the zero-point energy correction for all structures was calculated at the B3LYP level; see the SI for further details and benchmarking. The wave function stabilization, i.e., testing the stability of the single-determinant wave function with respect to lifting various constraints, was performed prior to each single-reference calculation. This procedure is very important in the case of transition metals due to the possible presence of multitude of electronic states, and its usage restricts the number of electronic structure codes that might be applied for the present calculations. In Table S4, we include comparison between CCSD, CCSD(T), and multireference configuration interaction (MRCI) with the Davidson correction for three local minima along the reaction pathway, showing that semiquantitative agreement between both methods is reached. The usage of MRCI for all investigated structures (which would be desirable) is complicated due to the need to preserve the active space composition along the whole pathway as well as the size-inconsistency of the method. The aug-cc-pVTZ basis set was used for C and O atoms, the ECP60MDF-AVTZ basis set was used for Ta.^57^ The minimum energy crossing points (MECPs) were searched at the CCSD level using the EasyMECP program^58^ that was modified to allow for performing wave function stabilization prior to each CCSD calculation. The relative position of MECPs compared to that of the ^3^[TaCO_2_]^+^ transition state was calculated at the CCSD level (see Table S5 for further details). For the ^1^Ta^+^, wave function stabilization was not performed as it predicted alow electronic energy, most probably due to multireference character of the species. The Gaussian package was used for single-reference calculations,^59^ and the Molpro program was used for multireference ones.^60^

### Hazards

No hazards are associated with the present study.

## Supporting Information Available

Figure S1: Comparison of experimental energy distributions for θ = 0° – 180° and the indicated pie cut θ = 0° – 160°. Figure S2: Differential cross sections with kinematic cut-offs for the reaction of ^5^Ta^+^ to ^5^TaO^+^ and to ^3^TaO^+^ superimposed for comparison. Figure S3: Product kinetic energy distributions *E*_rel_^′^. Tab. S1: Mean energies of the respective experimental energy distributions. Tab. S2 State distribution for thermal plasma. Tab. S3: Relative energies of stationary points. Tab. S4: Relative energies of three structures using MRCI, CCSD and CCSD(T). Tab. S5: Energies of the minimum energy crossing points (MECPs). Tab. S6: Energies of stationary points as optimized at the CCSD and CCSD(T) level. Tab. S7: Energies of stationary points optimized at B3LYP level. Cartesian coordinates of optimized structures (PDF)
