# Supplementary material for: Dynamics of the Oxygen Atom Transfer Reaction between Carbon Dioxide and the Tantalum Cation
Source: J Phys Chem Lett. 2023 Jun 8;14(24):5524–30. doi: 10.1021/acs.jpclett.3c01078 (PMC10291641; doi:10.1021/acs.jpclett.3c01078)
Supplement: Supplementary file 1 — jz3c01078_si_001.pdf [file jz3c01078_si_001.pdf]

# Dynamics of the Oxygen Atom Transfer Reaction between Carbon Dioxide and the Tantalum Cation
# Supplementary Information

Marcel Meta,† Maximilian E. Huber,† Tim Michaelsen,‡ Atilay Ayasli,‡ Milan987Ončák,‡ Roland Wester,‡ and Jennifer Meyer*,†

†*Fachbereich Chemie und Forschungszentrum OPTIMAS, RPTU Kaiserslautern-Landau, Erwin-Schrödinger Straße 52, 67663 Kaiserslautern, Germany*

‡*Institut für Ionenphysik und Angewandte Physik,Universität Innsbruck, Technikerstraße 25, 6020 Innsbruck, Austria*

E-mail: jmeyer@chemie.uni-kl.de

Phone: +49 663 205 4211

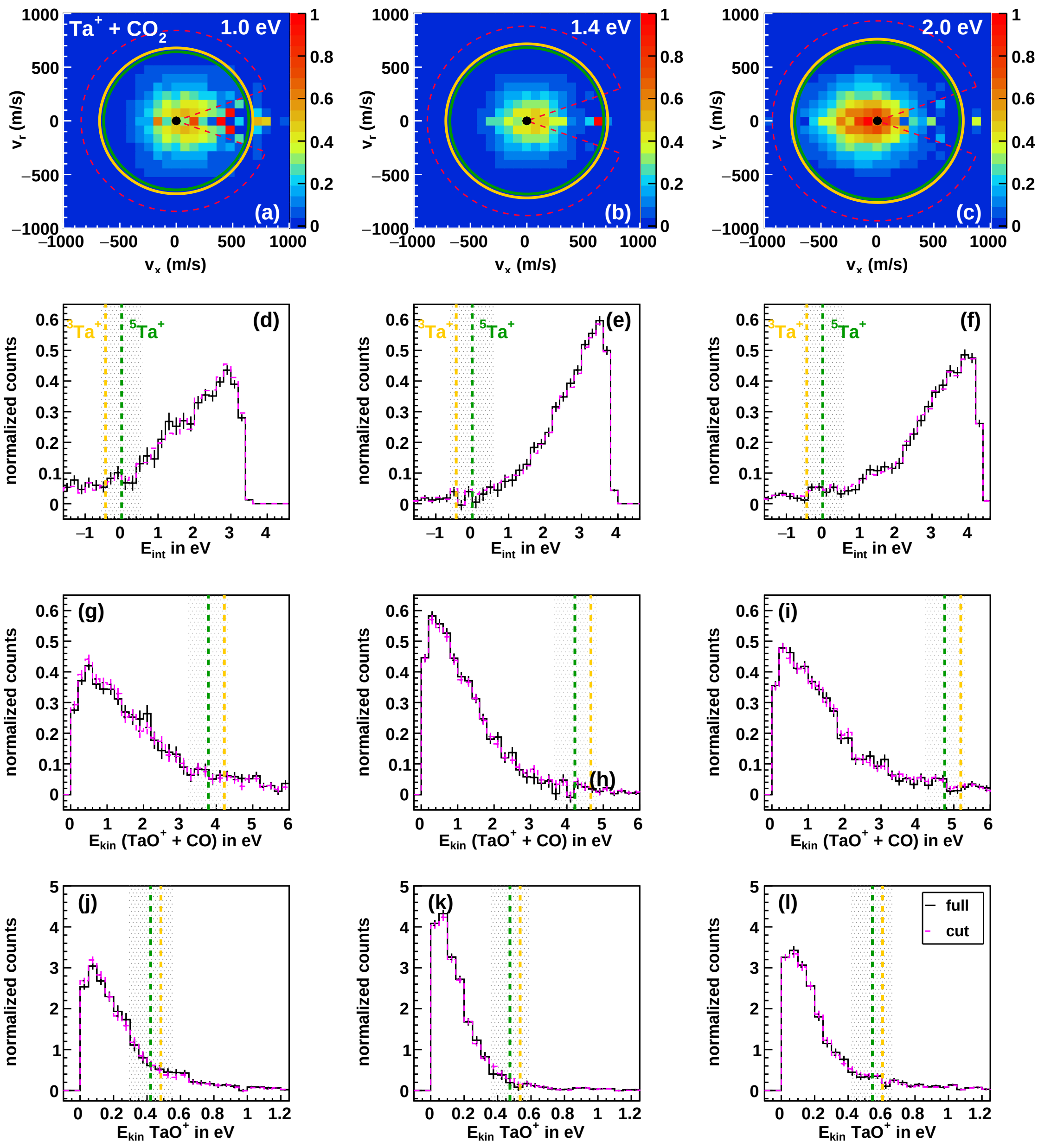

Figure S1: **Experimental energy distributions**. (a-c) Velocity distributions of the $TaO^+$ product ion at three collision energies in the range from 1–2 eV The superimposed circles represent the kinematic cut-off for reaction of $Ta^+$ in its electronic ground state ($^5Ta^+$, green) and the first electronically excited state ($^3Ta^+$, orange). At a scattering angle at or close to $\theta = 180°$, residual hits of the incident ion beam are visible. Internal (d-f), product kinetic (g-i) and product ion $TaO^+$ kinetic energy distributions are shown. Black lines give the integrated energy distributions over the whole scattering angle $\theta = 0° - 180°$ and pink the angular range without the ion beam incidences as indicated by the pink pie cut $\theta = 0° - 160°$ in the first row. The shaded area around the respective kinematic cuf-off for the ground state represent the error in the energy distributions after Gaussian error propagation from the reactant beam uncertainties

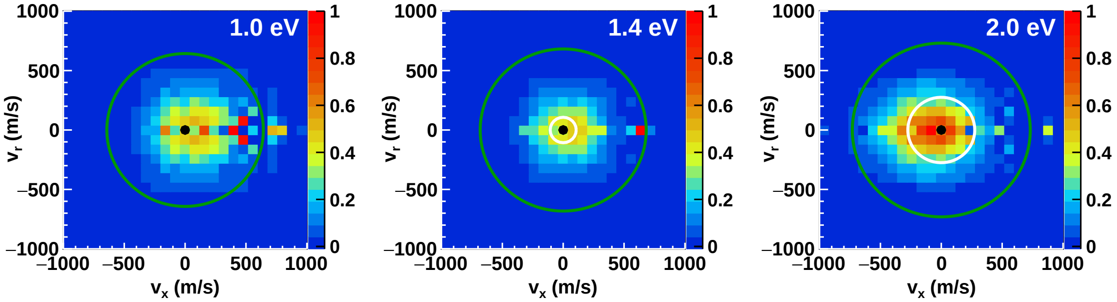


Figure S2: **Experimental differential cross sections**. Superimposed on the the scattering distributions are the kinematic cut-offs for reactions of $Ta^+$ in the quintet state to $TaO^+$ in the triplet state (green) and as comparison to the quintet state (white) which is endothermic by 1.34 eV. At the lowest collision energy, the endothermic channel is not open.

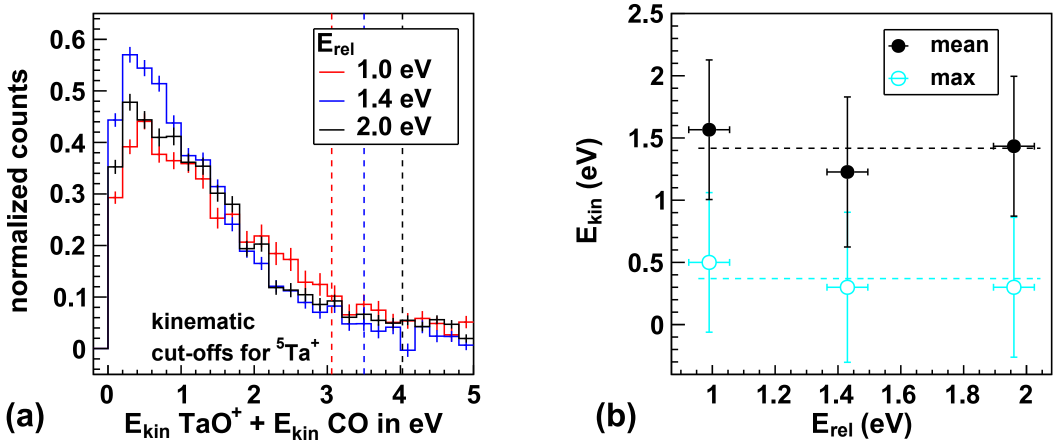


Figure S3: **Product kinetic energy distributions.** Panel (a) gives a comparison of the energy partitioned product kinetic energy, i. e. into the motion of the $TaO^+$ and CO at the three investigated collision energies. Panel (b) gives the means of the respective distributions (black, closed circle) and the bin with the maximum intensity (cyan, open circle). The kinetic energy distributions are almost the same at all three collision energies. The dashed lines in (b) are fits to data. The error bars illustrate $1\sigma$-errors of the respective energy distributions as by Gaussian error propagation from the input beams. All distributions are calculated from the hits within a pie cut as indicated by the pink dashed lines in figure 2, which lack contribution from the residual ion beam. In figure S1, comparisons to the full distributions are given.

Table S1: **Mean energies of the respective energy distributions** Means are calculated from distributions shown in figure 3 and figure S2. The total available energy given by the relative collision energy $E_{\mathrm{rel}}$ plus reaction from $^5Ta^+ + CO_2 \rightarrow ^3 TaO^+ + CO$.

| $E_{\mathrm{rel}}$ (eV) | mean $E_{\mathrm{kin}}$ (eV) | mean $E'_{\mathrm{rel}}$ (eV) | mean $E_{\mathrm{int}}$ (eV) |
|---|---|---|---|
| 0.99 | 0.26 | 1.57 | 1.83 |
| 1.43 | 0.17 | 1.23 | 2.58 |
| 1.96 | 0.23 | 1.43 | 2.83 |

Table S2: **Electronic states of $Ta^+$.** The calculation of the populated electronic states of $Ta^+$ have been simulated with a Maxwell-Boltzmann statistic.

| State | J | Electron configuration | Energy[a] (eV) | Population[b] (%) | | | | | |
|---|---|---|---|---|---|---|---|---|---|
| | | | | $\sum$ | 300 K | 600 K | 1000 K | 2000 K | 3000 K |
| $a^5F$ | 1 | $6s^1 5d^3$ | 0.000 | | 98.83 | 87.29 | 68.73 | 38.96 | 25.39 |
| | 2 | | 0.128 | | 1.17 | 12.27 | 25.98 | 30.92 | 25.81 |
| | 3 | | 0.328 | | 0.00 | 0.36 | 3.58 | 13.59 | 16.68 |
| | 4 | | 0.547 | | 0.00 | 0.01 | 0.36 | 4.88 | 9.16 |
| | 5 | | 0.767 | | 0.00 | 0.00 | 0.03 | 1.67 | 4.79 |
| $a^3F$ | 2 | $6s^2 5d^2$ | 0.394 | | 0.00 | 0.07 | 1.18 | 6.59 | 9.21 |
| | 3 | | 0.847 | | 0.00 | 0.00 | 0.01 | 0.67 | 2.24 |
| | 4 | | 1.208 | | 0.00 | 0.00 | 0.00 | 0.11 | 0.71 |
| $a^3P$ | 0 | $6s^2 5d^2$ | 0.511 | | 0.00 | 0.00 | 0.06 | 0.67 | 1.17 |
| | 1 | | 0.661 | | 0.00 | 0.00 | 0.03 | 0.84 | 1.97 |
| | 2 | | 0.702 | | 0.00 | 0.00 | 0.03 | 1.11 | 2.81 |
| $a^1D$ | 2 | $6s^2 5d^2$ | 1.681 | | 0.00 | 0.00 | 0.00 | 0.00 | 0.06 |
| | | | | $a^5F$ | 100.00 | 99.93 | 98.68 | 90.14 | 81.83 |
| | | | | $a^3F+a^3P$ | 0.00 | 0.07 | 1.32 | 9.98 | 18.10 |
| | | | | $a^1D$ | 0.00 | 0.00 | 0.00 | 0.00 | 0.06 |

[a]Atomic Spectra Database: https://nvlpubs.nist.gov
[b]Maxwell-Boltzmann distribution

Table S3: **Energies of stationary points using the computational approach described in the main text**. All values given relative to $^5Ta^+ + CO_2$

| | Energy (eV) | | |
|---|---|---|---|
| | quintet | triplet | singlet |
| $Ta^+ + CO_2$ | 0.00 | 0.39 | 1.33 |
| Pre-reaction well (LM1) $[TaCO_2]^+$ | -0.96 | -0.62 | -0.45 |
| Crossing point (MECP) | | -0.47 | -0.45 |
| Transition state (TS) | 0.53 | -0.34 | -0.07 |
| Post-reaction well (LM2) $[OTaCO]^+$ | -0.16 | -3.91 | -3.68 |
| $TaO^+ + CO$ | 1.34 | -2.40 | -2.28 |

Table S4: **Relative energy (in eV) in three structures optimized at the CCSD level in different spin multiplicities using different computational treatments.** Within the MRCI method, the active spaces of (4,9), (8,9) and (8,11) were used for $[TaCO_2]^+$, $[OTaCO]^+$, and $TaO^+$, respectively; the Davidson correction was applied. The zero-point energy correction was not applied.

| minimum | spin multiplicity | MRCI | CCSD | CCSD(T) |
|---|---|---|---|---|
| $^5[TaCO_2]^+$ | quintet | 0.00 | 0.00 | 0.00 |
| | triplet | 0.29 | 0.47 | 0.36 |
| | singlet | 0.78 | 0.67 | 0.52 |
| $^3[OTaCO]^+$ | triplet | 0.00 | 0.00 | 0.00 |
| | singlet | 0.58 | 0.30 | 0.23 |
| $^3[TaO]^+$ | triplet | 0.00 | 0.00 | 0.00 |
| | singlet | 0.31 | 0.21 | 0.13 |

Table S5: **Energies (in eV) of triplet/quintet and singlet/quintet minimum energy crossing points as optimized at the CCSD level and single-point recalculated at the CCSD(T) level.** Energies are given with respect to the triplet transition state as optimized at the B3LYP level and re-calculated at the CCSD or CCSD(T) level

| | CCSD | CCSD(T) |
|---|---|---|
| triplet/quintet | -0.13/-0.13 | -0.19/-0.10 |
| singlet/quintet | -0.12/-0.12 | -0.32/-0.09 |

Table S6: **Energies of stationary points at Coupled Cluster level.** Energies (in eV) of stationary points as optimized at the CCSD/aug-cc-pVTZ,ECP60MDF-AVTZ level along with a final re-calculation at the CCSD(T)/aug-cc-pVTZ,ECP60MDF-AVTZ level and the zero-point energy calculated at the CCSD/aug-cc-pVDZ,ECP60MDF-AVDZ level. For transition states, structures optimized at the CCSD/aug-cc-pVDZ,ECP60MDF-AVDZ level were used. The transition state for the triplet state multiplicity could not be optimized due to electronic structure issues.

| | Energy (eV) | | |
|---|---|---|---|
| | quintet | triplet | singlet |
| $Ta^+ + CO_2$ | 0.00 | 0.39 | 1.33 |
| pre-reaction well (LM1) $[TaCO_2]^+$ | -0.96 | -0.62 | -0.44 |
| Transition state (TS) | 0.54 | – | -0.03 |
| Post-reaction well (LM2) $[OTaCO]^+$ | -0.17 | -3.91 | -3.68 |
| $TaO^+ + CO$ | 1.34 | -2.40 | -2.28 |

Table S7: **Energies of stationary points at the B3LYP level**. Energies of stationary points as calculated at the B3LYP/aug-cc-pVTZ,ECP60MDF-AVTZ level along with the zero-point energy calculated at the at same level.

| | Energy (eV) | | |
|---|---|---|---|
| | quintet | triplet | singlet |
| $Ta^+ + CO_2$ | 0.00 | 0.37 | 1.92 |
| pre-reaction well (LM1) $[TaCO_2]^+$ | -0.89 | -0.57 | -0.35 |
| Transition state (TS) | 0.72 | -0.28 | -0.08 |
| Post-reaction well (LM2) $[OTaCO]^+$ | -0.08 | -3.50 | -3.31 |
| $TaO^+ + CO$ | 1.57 | -2.00 | -1.86 |

**Cartesian coordinates (in Angstrom) of optimized structures along with their electronic energies calculated at the CCSD(T) level (in Hartree)**

**$CO_2$**

$E = -0.18834038711\,\mathrm{D}+03$

| | | | |
|---|---|---|---|
| O | 0.000000 | 0.000000 | 1.159706 |
| C | 0.000000 | 0.000000 | 0.000000 |
| O | 0.000000 | 0.000000 | -1.159706 |

**CO**

$E = -0.11316208146\,\mathrm{D}+03$

| | | | |
|---|---|---|---|
| O | 0.000000 | 0.000000 | 0.483757 |
| C | 0.000000 | 0.000000 | -0.645009 |

**$^1[TaCO_2]^+$, LM1**

$E = -0.24515490618\,\mathrm{D}+03$

| | | | |
|---|---|---|---|
| Ta | 0.000000 | 0.000000 | 0.773828 |
| O | 0.000000 | 0.000000 | -1.403983 |
| C | 0.000000 | 0.000000 | -2.581972 |
| O | 0.000000 | 0.000000 | -3.720715 |

**$^3[TaCO_2]^+$, LM1**

$E = -0.24516126098\,\mathrm{D}+03$

| | | | |
|---|---|---|---|
| Ta | -0.000000 | 0.767394 | 0.000000 |
| O | -0.005692 | -1.381783 | -0.000000 |
| C | 0.000216 | -2.562154 | -0.000000 |
| O | 0.005530 | -3.699071 | -0.000000 |

**$^5[TaCO_2]^+$, LM1**

$E = -0.24517387022\,\mathrm{D}+03$

| | | | |
|---|---|---|---|
| Ta | 0.000000 | 0.000000 | 0.781095 |
| O | 0.000000 | 0.000000 | -1.428303 |
| C | 0.000000 | 0.000000 | -2.605491 |
| O | 0.000000 | 0.000000 | -3.745068 |

**$^1[TaCO_2]^+$, TS**

$E = -0.24513988938\,\mathrm{D}+03$

| | | | |
|---|---|---|---|
| Ta | 0.000000 | 0.615121 | 0.000000 |
| C | -0.092768 | -1.988796 | 0.000000 |
| O | 0.877512 | -1.243457 | 0.000000 |
| O | -0.807936 | -2.877929 | 0.000000 |

**$^3[TaCO_2]^+$, TS**

$E = -0.24514943215\,\mathrm{D}+03$

| | | | |
|---|---|---|---|
| Ta | 0.609790 | -0.056391 | -0.000009 |
| C | -1.971888 | 0.079000 | 0.001369 |
| O | -1.150158 | 0.989905 | -0.000387 |
| O | -2.935258 | -0.534589 | -0.000556 |

**$^5[TaCO_2]^+$, TS**

$E = -0.24511531798\,\mathrm{D}+03$

| | | | |
|---|---|---|---|
| O | 1.222385 | -0.961381 | 0.000000 |
| C | -0.521748 | -1.551519 | 0.000000 |
| O | -0.831074 | -2.638790 | 0.000000 |
| Ta | -0.000000 | 0.522061 | 0.000000 |

**$^{1}[TaCO_2]^+$, LM2**

$E = -0.24527146291\,\mathrm{D}+03$

| | | | |
|---|---|---|---|
| C | -0.731229 | -1.622668 | 0.000000 |
| O | -1.039651 | -2.699636 | 0.000000 |
| Ta | -0.000000 | 0.436313 | 0.000000 |
| O | 1.588073 | -0.064723 | 0.000000 |

**$^{3}[TaCO_2]^+$, LM2**

$E = -0.24527995251\,\mathrm{D}+03$

| | | | |
|---|---|---|---|
| C | -0.732850 | -1.636088 | 0.000000 |
| O | -1.036233 | -2.713350 | 0.000000 |
| Ta | 0.000000 | 0.439612 | 0.000000 |
| O | 1.585871 | -0.071041 | 0.000000 |

**$^{5}[TaO]^+$, LM2**

$E = -0.24514135110\,\mathrm{D}+03$

| | | | |
|---|---|---|---|
| C | -0.894391 | -1.586096 | 0.000000 |
| O | -1.227557 | -2.659879 | 0.000000 |
| Ta | 0.000000 | 0.372281 | 0.000000 |
| O | 1.898350 | 0.452385 | 0.000000 |

**$^{1}[TaO]^+$**

$E = -0.13205529880\,\mathrm{D}+03$

| | | | |
|---|---|---|---|
| Ta | 0.000000 | 0.000000 | 0.163231 |
| O | 0.000000 | 0.000000 | -1.489482 |

**$^{3}[TaO]^+$**

$E = -0.13205993745\,\mathrm{D}+03$

| | | | |
|---|---|---|---|
| Ta | 0.000000 | 0.000000 | 0.163335 |
| O | 0.000000 | 0.000000 | -1.490431 |

**$^{5}[TaO]^+$**

$E = -0.13192161468\,\mathrm{D}+03$

| | | | |
|---|---|---|---|
| Ta | 0.000000 | 0.000000 | 0.183479 |
| O | 0.000000 | 0.000000 | -1.674250 |

**$^{1}Ta^+$**

$E = -0.56748693540\,\mathrm{D}+02$

| | | | |
|---|---|---|---|
| Ta | 0.000000 | 0.000000 | 0.000000 |

**$^{3}Ta^+$**

$E = -0.56783043957\,\mathrm{D}+02$

| | | | |
|---|---|---|---|
| Ta | 0.000000 | 0.000000 | 0.000000 |

**$^{5}Ta^+$**

$E = -0.56797496469\,\mathrm{D}+02$

| | | | |
|---|---|---|---|
| Ta | 0.000000 | 0.000000 | 0.000000 |

**MECP (singlet/quintet)**

$E = -0.24516103684\,\mathrm{D}+03, -0.24515268576\,\mathrm{D}+03$

| | | | |
|---|---|---|---|
| Ta | -0.539870 | -0.063431 | -0.000065 |
| C | 1.644577 | -0.007355 | 0.004328 |
| O | 1.017935 | 1.126398 | -0.000871 |
| O | 2.674944 | -0.542071 | -0.001784 |

**MECP (triplet/quintet)**

$E = -0.24515628440\,\mathrm{D}+03, -0.24515298286\,\mathrm{D}+03$

| | | | |
|---|---|---|---|
| Ta | -0.547861 | -0.062619 | 0.000000 |
| C | 1.679610 | -0.009265 | -0.000002 |
| O | 1.025194 | 1.107791 | 0.000000 |
| O | 2.714334 | -0.529441 | 0.000001 |
